# KUSp, a novel oral spindle cell squamous cell carcinoma cell line with epithelial-mesenchymal transition features and high invasive capacity

**DOI:** 10.1007/s13577-026-01464-3

**Published:** 2026-11-07

**Authors:** Kenta Kawahara, Yusei Todoroki, Kazumasa Ohyumi, Junki Inoue, Koh Harada, Yuki Seki, Yutaro Fujimori, Mayuko Yamashita, Keisuke Yamana, Hikaru Nakashima, Akiyuki Hirosue, Masatoshi Hirayama, Ryoji Yoshida, Seiji Okada, Hideki Nakayama

**Affiliations:** 1 Department of Oral and Maxillofacial Surgery, Faculty of Life Sciences, Kumamoto University, Honjo 1-1-1, Chuo-ku, Kumamoto, 860-8556 Japan; 2 Division of Hematopoiesis, Joint Research Center for Human Retrovirus Infection, Kumamoto University, Kumamoto, Japan

**Keywords:** Spindle cell squamous cell carcinoma, Epithelial–mesenchymal transition, Cell line, Cervical lymph node metastasis, Oral cancer

## Abstract

Spindle cell squamous cell carcinoma (SpSCC) is a rare and aggressive variant of squamous cell carcinoma characterized by biphasic histopathology and epithelial–mesenchymal transition (EMT)-associated features. The scarcity of well-characterized experimental models, particularly those derived from metastatic lesions, has limited investigation of its biology. Here, we established KUSp, a cell line derived from a cervical lymph node metastasis of oral SpSCC, and characterized its pathological, phenotypic, and functional features. The primary tumor showed conventional squamous and spindle cell components, whereas the metastatic lesion predominantly contained spindle-shaped tumor cells expressing both epithelial and mesenchymal markers. KUSp displayed a pronounced EMT-like phenotype characterized by a spindle-shaped morphology, loss of cytokeratin AE1/AE3 and E-cadherin expression, and expression of vimentin, N-cadherin, and α-smooth muscle actin. Functionally, KUSp showed significantly greater Matrigel invasion than the established oral SCC cell lines examined, including HOC313, together with robust migratory activity. These findings characterize KUSp as a patient-derived in vitro model combining pronounced EMT-associated features with high invasive capacity and robust migratory activity, providing a resource for investigating the relationship between EMT-associated phenotypes and invasive behavior in metastatic oral SpSCC.

**Supplementary Information:**

The online version contains supplementary material available at https://doi.org/10.1007/s13577-026-01464-3.

## Introduction

Spindle cell squamous cell carcinoma (SpSCC) is a rare and aggressive variant of squamous cell carcinoma (SCC) characterized by biphasic histopathology, including both epithelial and spindle cell (sarcomatoid) components [1–5]. According to the current World Health Organization classification of head and neck tumors and the International Classification of Diseases, 11th Revision for mortality and morbidity statistics, SpSCC is categorized as a histological subtype of SCC of the oral cavity [1, 6, 7]. The spindle cell phenotype is hypothesized to emerge through epithelial–mesenchymal transition (EMT), rendering SpSCC a significant tumor model for studies related to EMT [8]. SpSCC accounts for < 1% of oral cancers and poses a considerable diagnostic challenge owing to its morphological heterogeneity and similarity to other spindle cell tumors [1–5]. Furthermore, the spindle-cell morphology and mesenchymal features of SpSCC can complicate its distinction from other spindle-cell lesions, particularly when epithelial marker expression is reduced; therefore, careful characterization of cells established in primary culture is required [2, 4]. Clinically, SpSCC is associated with rapid growth, frequent local recurrence, and a poor prognosis [3, 9, 10]. However, advancements in the understanding of SpSCC have been impeded by the limited availability of reliable in vitro models [5, 11]. Recently, only a few cell lines have been established, primarily from primary lesions [5, 11], with virtually no cell lines available from metastatic sites. The development of new SpSCC cell lines, particularly from metastatic disease, is crucial for elucidating the molecular mechanisms of tumor progression and evaluating potential therapeutic agents.

In this study, we aimed to establish and characterize a novel SpSCC cell line derived from cervical lymph node metastasis of oral cancer. We evaluated its pathological and EMT-associated features, proliferative, invasive, and migratory properties, and genetic relationship with the corresponding primary and metastatic tumor tissues. We also assessed its tumor-forming potential in immunodeficient mice to define the experimental utility and limitations of this cell line.

## Materials and methods

### Case history

**Fig. 1:**
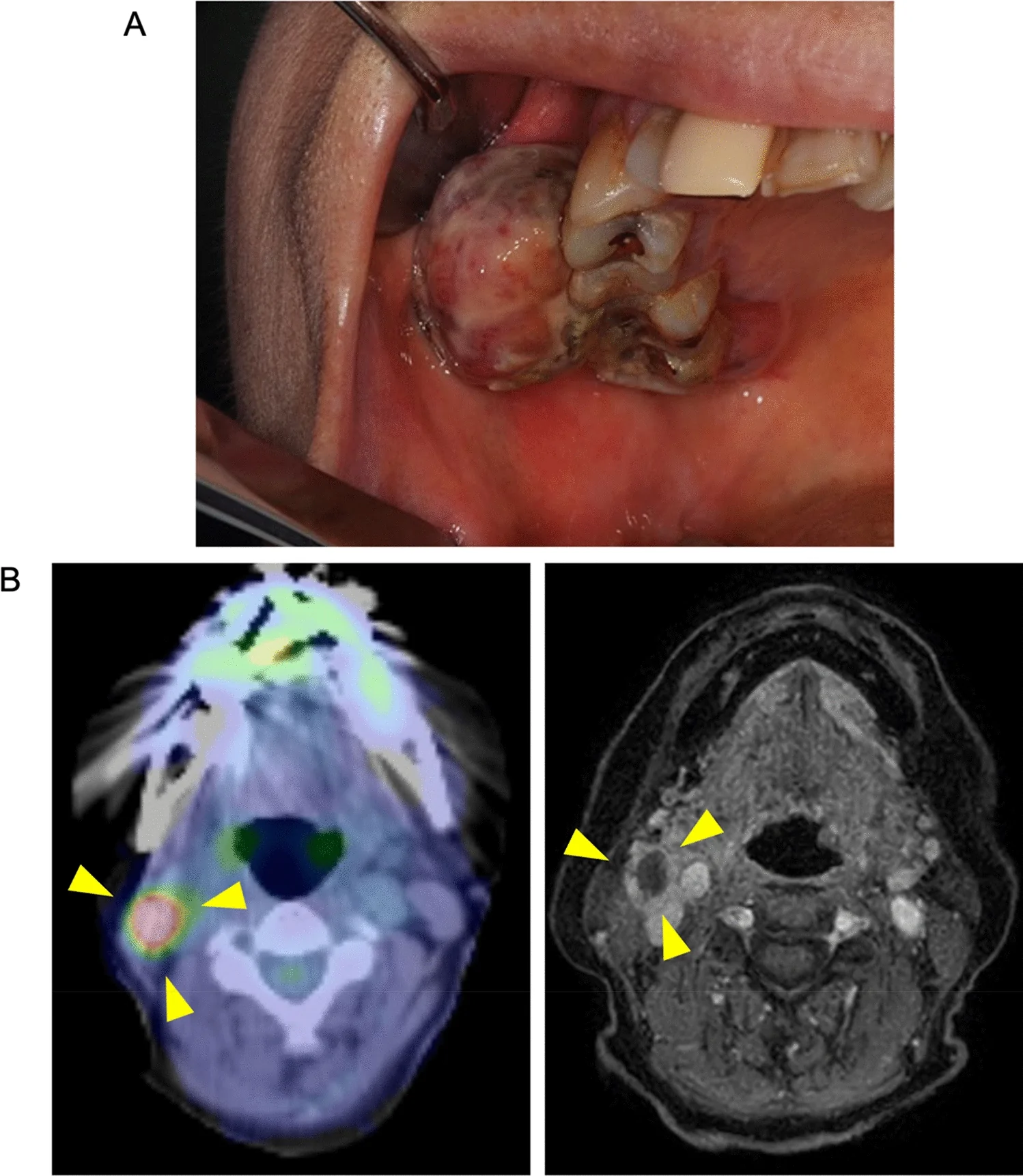
Clinical presentation and imaging of the patient. **A** Intraoral photograph showing an exophytic, ulcerated tumor on the right maxillary gingiva (arrow). **B** Imaging findings at the time of detection of cervical lymph node metastasis. Left: Positron emission tomography-computed tomography (PET-CT) demonstrating FDG uptake in the right posterior cervical lymph node (arrow). Right: Contrast-enhanced magnetic resonance imaging (MRI) revealing an enlarged right cervical lymph node (arrow)

An 84-year-old Japanese woman was referred to our department in 2016 for evaluation of a neoplastic lesion of the right maxilla. A biopsy specimen was histopathologically diagnosed as spindle cell squamous cell carcinoma (SpSCC). Under general anesthesia, partial resection of the right maxilla was performed for SpSCC of the right maxillary gingiva (Fig. 1A). The final pathological stage was pT4aN0M0 according to the UICC TNM Classification, 8th edition. Approximately 8 months after surgery, metastasis to the right posterior cervical lymph node was detected by imaging examinations (Fig. 1B), and a modified radical neck dissection was performed. Tumor tissue from the metastatic lymph node was used to establish the KUSp cell line. The patient had not received radiotherapy or chemotherapy before tissue collection.

This study was conducted in accordance with the ethical standards of the relevant institutional and/or national research committee and the 1964 Declaration of Helsinki and its later amendments. Written informed consent for the use of tumor tissue and clinical information for research was obtained from the patient. The study was approved by the Ethics Committee of Kumamoto University (RINRI No. 1928; Genome No. 588).

### Establishment of cell line

Tumor tissue was obtained from a metastatic right posterior cervical lymph node resected during modified radical neck dissection and was processed immediately after surgery. Blood and macroscopically necrotic tissue adhering to the specimen were carefully removed under sterile conditions. The remaining tumor tissue was washed thoroughly with phosphate-buffered saline (PBS), minced into small fragments, and placed directly into culture dishes for primary explant culture. The tissue fragments were cultured in Dulbecco’s Modified Eagle Medium (DMEM; D6429, Sigma-Aldrich, St. Louis, MO, USA) supplemented with 10% fetal bovine serum (FBS; Sigma-Aldrich) and penicillin–streptomycin (Gibco, Thermo Fisher Scientific, Waltham, MA, USA) at 37 °C in a humidified atmosphere containing 5% CO_2_. The established cell line was designated KUSp.

### Cell lines and culture

Human oral SCC (OSCC) cell lines derived from SAS tongue and Ca9-22 gingival tumors were obtained from the RIKEN BioResource Center (Ibaraki, Japan). The HOC313 cell line was kindly donated by the Department of Oral and Maxillofacial Surgery, Kanazawa University Graduate School of Medical Science. The human oral fibroblast cell line GT-1 was kindly provided by Professor Kamata (Hiroshima University, Hiroshima, Japan), as previously described [12]. The cell lines were cultured with DMEM (D6429; Sigma-Aldrich, Saint Louis, MO, USA) supplemented with 10% FBS and maintained under humidified 5% CO_2_ incubation at 37 °C. For RT-qPCR analysis, GT-1 cells were treated with recombinant human TGF-β1 (10 ng/mL) for 72 h to generate CAF-like cells, as previously described [12].

### Short tandem repeat profiling and cell line authentication

To confirm the patient origin of KUSp and assess the identity of the comparator cell stocks, short tandem repeat (STR) profiling was performed by BEX Corporation (Tokyo, Japan) using the PowerPlex 16 HS System (Promega, Madison, WI, USA), according to the manufacturer's instructions. Genomic DNA was prepared from the primary tumor, cervical lymph node metastasis, KUSp cells, an archived HOC313 stock used in the experiments, and a separately maintained HSC-2 stock. The STR profile of KUSp was compared with those of the primary tumor and cervical lymph node metastasis to assess their common patient origin. The HOC313 and HSC-2 profiles were compared with each other and with those of KUSp and the two tumor specimens. In addition, the STR profile of KUSp was compared with reference human cell-line profiles in the Cellosaurus database using the CLASTR/Cellosaurus STR similarity search [13]. An evaluation value (EV) of ≥ 0.8 was considered indicative of a match consistent with a common donor origin [14].

### Cell proliferation assay

The cells were seeded in 96-well plates at a density of 2.0 × 10^3^ cells per well in sextuplicate and incubated in DMEM supplemented with 10% FBS for 24–120 h. Cell proliferation was assessed using the Cell Counting Kit-8 (Dojindo, Kumamoto, Japan), according to the manufacturer’s instructions. Briefly, the Cell Counting Kit-8 reagent was added to each well at a 1:10 dilution, and the plates were incubated for an additional 1–2 h at 37 °C. Absorbance was measured at 450 nm using an iMark Microplate Reader (Bio-Rad Laboratories, CA, USA).

### Immunohistochemistry

Formalin-fixed specimens were embedded in paraffin, cut into 4 μm sections, and mounted on MAS-GP-coated slides (Matsunami Glass Ind., LTD, Osaka, Japan). After deparaffinization and rehydration, the sections were heated in an autoclave in 10 μmol/L citrate buffer (pH 6.0) for 15 min at 121 °C for antigen retrieval. Subsequently, the sections were incubated with 3% H_2_O_2_ in absolute methanol for 30 min to block endogenous peroxidase. Subsequently, non-specific background staining was blocked using Protein Block Serum Free Reagent (Dako, Glostrup, Denmark) for 15 min, followed by overnight incubation at 4 °C with anti-cytokeratin AE1/AE3 (1:100 dilution, sc-81714, Santa Cruz), anti-E-cadherin (1:400 dilution, 610181, BD Biosciences), anti-vimentin (1:300 dilution, #5741, Cell Signaling Technology), anti-N-cadherin (1:50 dilution, sc-59987, Santa Cruz), and anti-α-smooth muscle actin (SMA) (1:200 dilution, M0851, Dako) antibodies diluted in PBS containing 1% bovine serum albumin (BSA). This was followed by sequential 60-min incubations with secondary antibodies (EnVision+ System-HRP Labeled Polymer; Dako) and visualization with the Liquid DAB+ Substrate Chromogen System (Dako). All slides were lightly counterstained with hematoxylin–eosin for 30 s before dehydration and mounting.

### Adipogenic and osteogenic differentiation

The adipogenic and osteogenic differentiation capacities of KUSp cells were assessed using the Human Mesenchymal Stem Cell Functional Identification Kit (SC006; R&D Systems) according to the manufacturer’s instructions. Adipose-Derived Mesenchymal Stem Cells (AD-MSCs) (PCS-500-011; ATCC) served as a positive control. For adipogenic differentiation, cells were seeded at 2.1 × 10^4^ cells/cm^2^ and cultured to confluence before induction. For osteogenic differentiation, cells were seeded at 4.2 × 10^3^ cells/cm^2^ and induced at 50–70% confluence. Differentiation media were renewed every 3–4 days. After 21 days, cells were fixed with 4% paraformaldehyde and stained for FABP4 or osteocalcin to assess adipogenic or osteogenic differentiation, respectively. Nuclei were counterstained with Hoechst 33,342.

### Immunofluorescence staining

The cells (2 × 10^4^) were seeded onto glass slides and incubated in DMEM with 10% FBS for 24 h. The cells were fixed in 4% paraformaldehyde in a PBS solution for 10 min and washed with PBS with Tween 20 (PBS-T). Transmittance processing was conducted with PBS-T containing 0.2% TritonX-100. Subsequently, the cells were washed with PBS-T and blocked with PBS-T containing 1% BSA and 0.15% glycine for 60 min. After blocking, the cells were incubated overnight at 4 °C for 24 h and then with anti-cytokeratin AE1/AE3 (1:100 dilution, sc-81714, Santa Cruz), anti-E-cadherin (1:100 dilution, 610181, BD Biosciences), anti-vimentin (1:100 dilution, #5741, Cell Signaling Technology), anti-N-cadherin (1:100 dilution, sc-59987, Santa Cruz), and anti-α-SMA (1:100 dilution, M0851, Dako) in PBS-T containing 0.1% BSA at 4 °C overnight. Subsequently, the glass slides were washed with PBS-T. The cells were incubated for 1 h at room temperature with secondary antibodies (Alexa Fluor 488 and 594 donkey anti-rabbit and/or anti-mouse IgG; Life Technologies) in PBS-T containing 0.1% BSA. After washing with PBS, the cells were counterstained and mounted using Vectashield (Vector Laboratories Inc) and observed under a fluorescent microscope (model BZ-X700; Keyence Corporation).

### Western blotting analysis

Whole cell proteins were separated using 10% sodium dodecyl sulfate-polyacrylamide gel electrophoresis, transferred onto nitrocellulose membranes, and probed with anti-cytokeratin AE1/AE3 (1:200 dilution, sc-81714, Santa Cruz), anti-E-cadherin (1:1000 dilution, 610181, BD Biosciences), anti-vimentin (1:1000 dilution, #5741, Cell Signaling Technology), anti-N-cadherin (1:200 dilution, sc-59987, Santa Cruz), and β-actin (1:5000 dilution; Sigma). After overnight incubation, the membranes were again washed three times for 10 min per wash in TBS-T, after which they were developed using an ECL Prime Detection Kit (GE Healthcare). The emitted light was measured using a C-DiGit Blot Scanner (LI-COR Biosciences), and the captured images were analyzed using Image Studio for C-Digit (LI-COR Biosciences).

### Cell invasion assay

The cell invasion assay was performed using BioCoat Matrigel Invasion Chambers (24 wells, 8 μm pore size [Corning]). Briefly, the cells were seeded at a density of 5 × 10^4^ cells per chamber and cultured in serum-free DMEM inside the chamber. The outside of the chamber was filled with DMEM supplemented with 10% FBS. After 48 h, the cells were fixed and stained using a Diff-Quick Stain Kit (Sysmex). The stained membranes of the chambers were mounted on slides, covered, and observed with a BX51 microscope (Olympus) using the CellSens standard program (version 1.16). Three fields of view were captured at random and analyzed using ImageJ version 1.49 [15].

### Scratch wound healing assay

The cell migration ability was determined using a scratch wound healing assay. The cells (5 × 10^4^ cells/ml) were seeded in 6-well plates and incubated at 37 °C until they were subconfluent. The monolayered cells were wounded by scratching with pipette tips and incubated further at 37 °C in DMEM supplemented with 0.5% FBS for 48 h. Phase contrast images of the cells were captured at the time of the scratching and afterward during the incubation using a CKX53 microscope (Olympus) equipped with the CellSens standard program (version 1.16). The degree of cell migration into the wounded area was calculated as the remaining space using ImageJ version 1.49 [15].

### RNA extraction and RT-qPCR analysis

Total RNA was extracted using the FastGene^™^ RNA Basic Kit (NIPPON Genetics), and RNA quantity and purity were assessed using a NanoDrop spectrophotometer (Thermo Fisher Scientific). cDNA was synthesized using the ReverTra Ace qPCR RT Master Mix with gDNA Remover (TOYOBO). RT-qPCR was performed using THUNDERBIRD Probe qPCR Mix (TOYOBO) on a ViiA^™^ 7 Real-Time PCR System (Thermo Fisher Scientific). The cycling conditions were 95 °C for 5 min, followed by 40 cycles of 95 °C for 15 s and 60 °C for 45 s. Expression levels were normalized to GAPDH and calculated using the 2^−ΔΔCt^ method. Primer sequences are listed in Supplementary Table S1.

### Whole-exome sequencing and comparative variant analysis

Genomic DNA from the KUSp cell line and primary tumor tissue (Primary) was extracted using the DNeasy Blood & Tissue Kit (Qiagen, Hilden, Germany) according to the manufacturer’s instructions. Genomic DNA from formalin-fixed, paraffin-embedded (FFPE) metastatic lymph node tissue (Meta) was extracted using the QIAamp DNA FFPE Tissue Kit (Qiagen, Hilden, Germany) according to the manufacturer’s instructions. Whole-exome sequencing (WES) was performed by Rhelixa Inc. (Tokyo, Japan). Detailed procedures for WES and comparative variant analysis are provided in Appendix S1.

### In vivo tumorigenicity assay

To evaluate the in vivo tumorigenic potential of KUSp cells, xenograft experiments were performed using nude mice and BALB/c-Rag2^−/−^Jak3^−/−^ (BRJ) mice [16]. KUSp cells (5 × 10^6^ cells per mouse) were mixed with Matrigel and inoculated into the dorsal subcutaneous tissue, tongue, or buccal mucosa. Five mice were used for each experimental condition. Mice were monitored for 4 weeks after inoculation for the development of palpable and progressively growing tumors. At the end of the observation period, the inoculation sites were examined macroscopically. All animal experiments were approved by the Ethics Committee for Animal Experiments of Kumamoto University (approval no. A2024-032) and performed in accordance with the guidelines for animal experiments in the laboratories.

### Statistical analyses

Statistical analyses were performed using EZR (Saitama Medical Center, Jichi Medical University, Saitama, Japan), a graphical user interface for R (The R Foundation for Statistical Computing, Vienna, Austria) and, more precisely, a modified version of R commander designed to add statistical functions frequently used in biostatistics [17]. Differences between the two groups were analyzed using the Wilcoxon rank-sum (Mann–Whitney) test. All P values were calculated based on two-tailed statistical analysis, and *P* value < 0.05 was considered statistically significant.

## Results

### Histopathological and immunohistochemical findings of original tumor

**Fig. 2:**
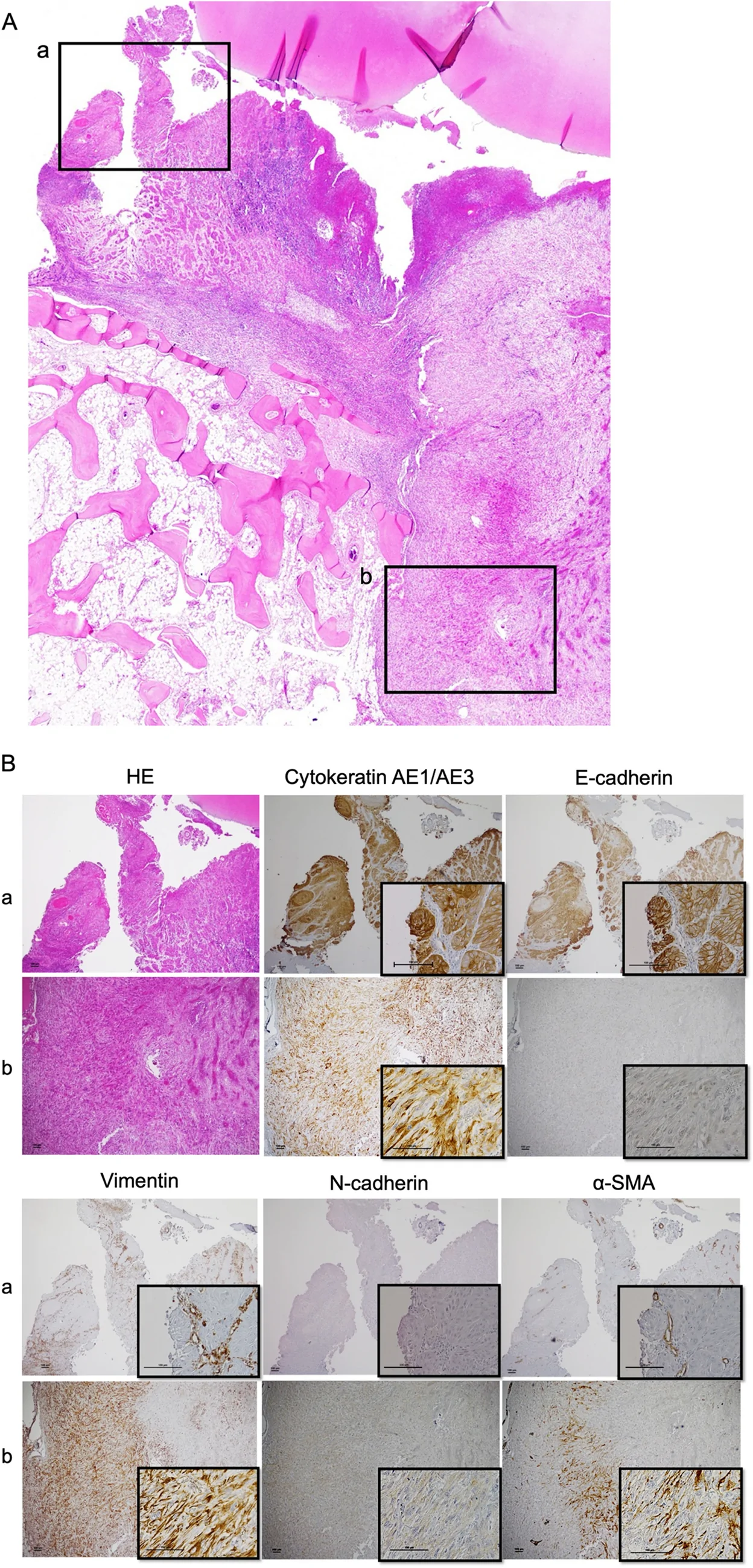

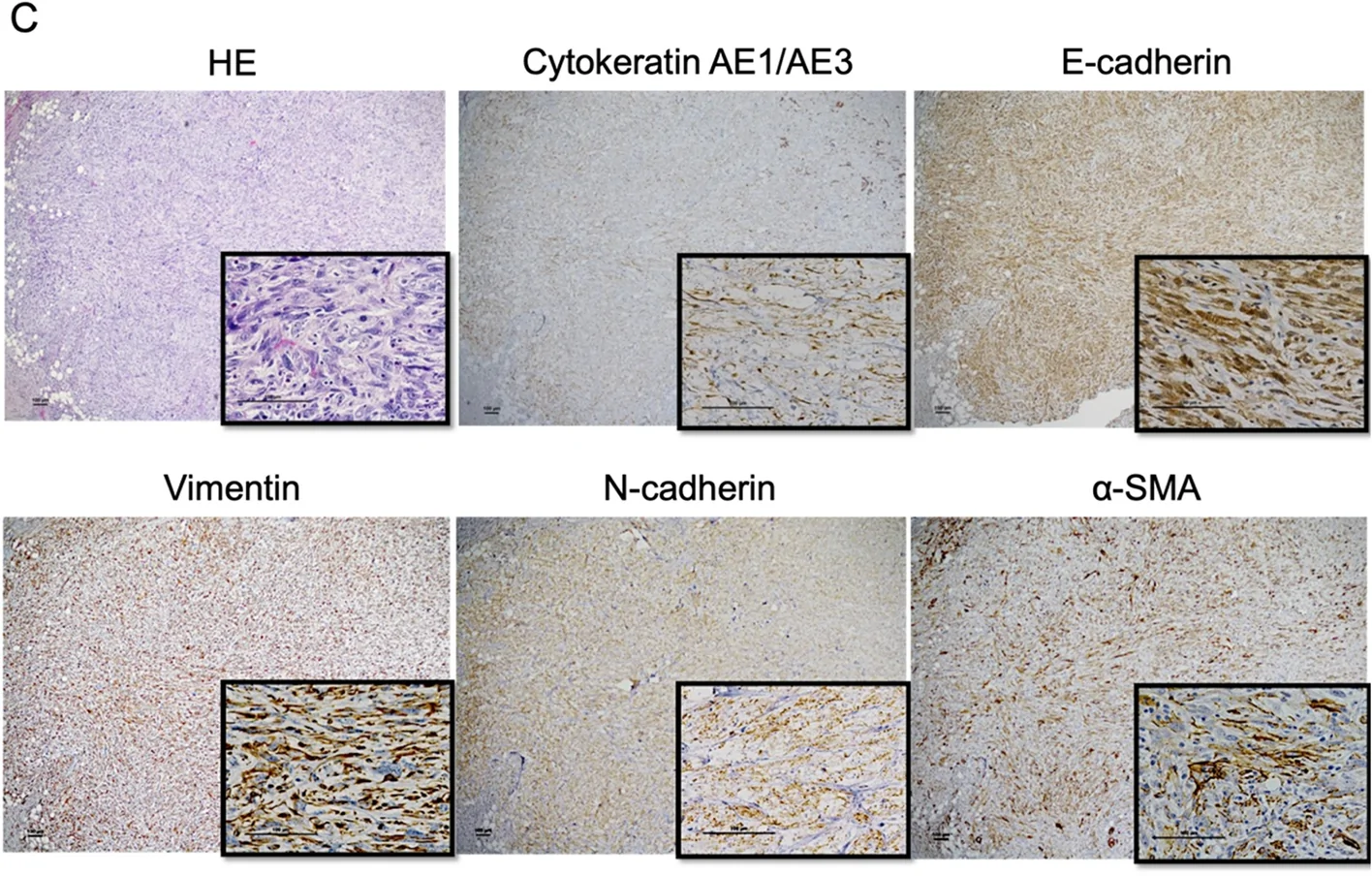
Histopathological and immunohistochemical analyses of the primary tumor and metastatic lymph node. **A** Hematoxylin and eosin (H&E) staining of the primary tumor reveals a biphasic morphology with a superficial conventional squamous cell carcinoma (SCC) component (**a**) and a deeper spindle cell squamous cell carcinoma (SpSCC) component (**b**). **B** Immunohistochemical staining of the primary lesion shows diffuse cytokeratin AE1/AE3 and E-cadherin positivity in the SCC area (**a**), whereas the spindle cell area (**b**) exhibits only focal or weak cytokeratin AE1/AE3 and E-cadherin expressions, with strong vimentin and α-SMA positivities and weak N-cadherin positivity, indicating a partial EMT-like phenotype. **C** In the metastatic cervical lymph node, spindle-shaped tumor cells display positive staining for E-cadherin, vimentin, N-cadherin, and α-SMA. Scale bar: 100 μm

Hematoxylin and eosin staining of the primary tumor is shown in Fig. 2A. The primary tumor consisted of an area of conventional SCC (Fig. 2A, a) and an area of SpSCC (Fig. 2A, b). Immunohistochemically, the SCC area was diffusely positive for cytokeratin AE1/AE3 and E-cadherin, whereas vimentin, N-cadherin, and α-SMA were negative (Fig. 2B, a). Meanwhile, the SpSCC area showed reduced cytokeratin AE1/AE3 and E-cadherin expressions and was strongly positive for vimentin and α-SMA, with weak positivity for N-cadherin (Fig. 2B, b), indicating a partial EMT-like phenotype within the spindle cell component. In the metastatic cervical lymph node, infiltration of spindle-shaped tumor cells was predominantly observed. Immunohistochemically, these cells were positive for cytokeratin AE1/AE3, E-cadherin, vimentin, N-cadherin, and α-SMA (Fig. 2C), suggesting that, at the time of lymph node metastasis, tumor cells had acquired a mesenchymal/EMT-like immunophenotype while still retaining epithelial characteristics. The overall immunohistochemical and immunofluorescence profiles of the primary tumor, cervical lymph node metastasis, and KUSp cells are summarized in Table 1.

**Table 1:** Immunohistochemical and immunofluorescence profiles of the primary oral spindle cell squamous cell carcinoma, cervical lymph node metastasis, and KUSp cell line

		AE1/AE3	E-cadherin	Vimentin	N-cadherin	α-SMA
Primary tumor	SCC	++	++	−	−	−
	SpSCC	+	+	++	+	++
Limph node metastasis		+	++	++	++	++
KUSp (IF)		−	−	++	++	++

*SCC* squamous cell carcinoma, *SpSCC* spindle cell squamous cell carcinoma, *AE1/AE3* cytokeratin AE1/AE3, *α-SMA* alpha-smooth muscle actin, *IF* immunofluorescence

−, negative; +, weakly positive; ++, strongly positive

### Establishment and characterization of a novel cell line derived from cervical lymph node metastasis

**Fig. 3:**
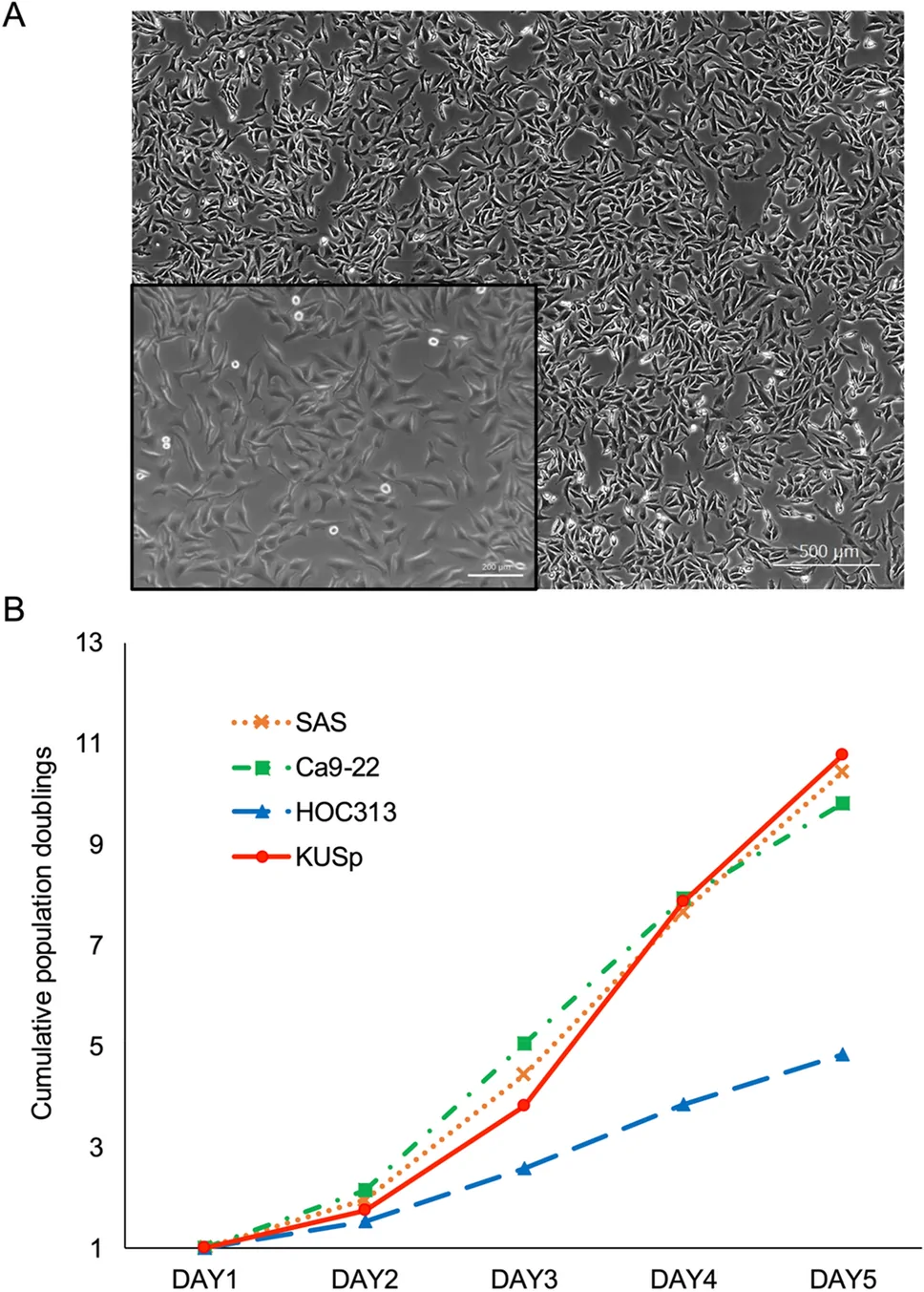
Establishment and growth characteristics of the KUSp cell line. **A** Representative phase-contrast micrograph of established KUSp cells derived from a metastatic cervical lymph node, showing a spindle-shaped morphology. The inset presents a higher-magnification view of the same field, highlighting the elongated cytoplasm and spindle-shaped nuclei. Scale bars, 500 µm in the main image and 200 µm in the inset. **B** Growth curves of KUSp and established oral squamous cell carcinoma (OSCC) cell lines (SAS, Ca9-22, HOC313). KUSp cells show a proliferation rate comparable with those of SAS and Ca9-22 and faster than that of HOC313. Cell proliferation was measured using the Cell Counting Kit-8 assay at the indicated time points

A continuously proliferating cell line, designated KUSp, was established and maintained for more than 100 passages. KUSp cells consistently exhibited a spindle-shaped morphology during serial passaging (Fig. 3A). To confirm the patient origin of KUSp and assess the identity of the comparator cell stocks, STR profiling was performed using DNA obtained from the primary tumor, metastatic cervical lymph node tumor, KUSp cells, HOC313 cells, and HSC-2 cells. The STR profile of KUSp was compatible with those of the corresponding primary and metastatic tumors. Repeat STR analysis of the archived HOC313 stock showed a profile distinct from that of HSC-2. Neither HOC313 nor HSC-2 met the predefined STR matching criterion (EV ≥ 0.8) in any pairwise comparison with KUSp, the primary tumor, or the metastatic cervical lymph node tumor (Supplementary Table S2). The growth rate of KUSp was comparable with that of SAS and Ca9-22 cells and faster than that of HOC313 cells (Fig. 3B).

### Differential expression of epithelial–mesenchymal transition markers in KUSp compared with conventional oral squamous cell carcinoma cell lines

**Fig. 4:**
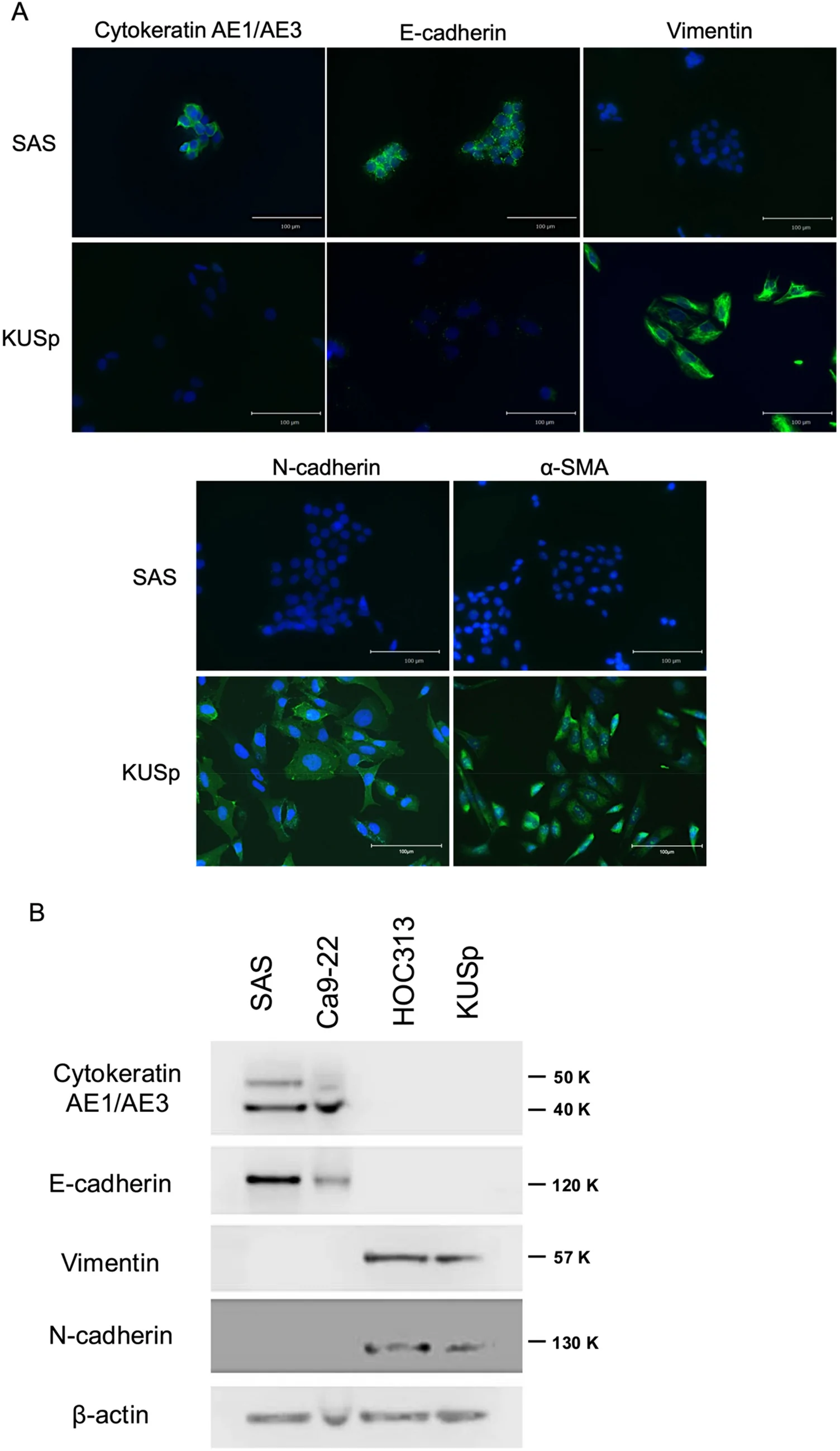
Differential expression of epithelial–mesenchymal transition (EMT) markers in KUSp and conventional OSCC cell lines. **A** Immunofluorescence staining of SAS and KUSp cells for epithelial markers (cytokeratin AE1/AE3, E-cadherin) and mesenchymal markers (vimentin, N-cadherin, α-SMA). SAS cells show strong expression of cytokeratin AE1/AE3 and E-cadherin, with little to no expression of mesenchymal markers. In contrast, KUSp cells are negative for cytokeratin AE1/AE3 and E-cadherin, but positive for vimentin, N-cadherin, and α-SMA. Nuclei are counterstained with DAPI. Scale bar: 100 µm. **B** Western blotting analysis of EMT marker expression in KUSp and conventional OSCC cell lines (SAS, Ca9-22, HOC313). SAS and Ca9-22 predominantly express epithelial markers (cytokeratin AE1/AE3, E-cadherin), whereas HOC313 and KUSp exhibit elevated levels of mesenchymal markers (vimentin, N-cadherin). β-actin was used as a loading control. Molecular mass markers are indicated on the left of the blots in K (kDa)

To clarify the expression patterns of epithelial and mesenchymal markers associated with EMT, immunofluorescence microscopy was performed. In SAS cells, epithelial markers cytokeratin AE1/AE3 and E-cadherin were clearly detected, whereas mesenchymal markers vimentin, N-cadherin, and α-SMA were not expressed. In contrast, KUSp cells showed positive expression of vimentin, N-cadherin, and α-SMA and were negative for cytokeratin AE1/AE3 and E-cadherin (Fig. 4A).

Furthermore, to compare EMT-related marker expression among different OSCC cell lines, western blotting analysis was performed. SAS and Ca9-22 predominantly expressed epithelial markers, including cytokeratin AE1/AE3 and E-cadherin, whereas HOC313 and KUSp exhibited elevated expression of mesenchymal markers, such as vimentin and N-cadherin (Fig. 4B). HOC313 is a well-characterized OSCC cell line that displays EMT-like properties [18–20], and the EMT-related marker expression profile of KUSp was similar to that of HOC313.

### Invasion and migration potential of KUSp cells

**Fig. 5:**
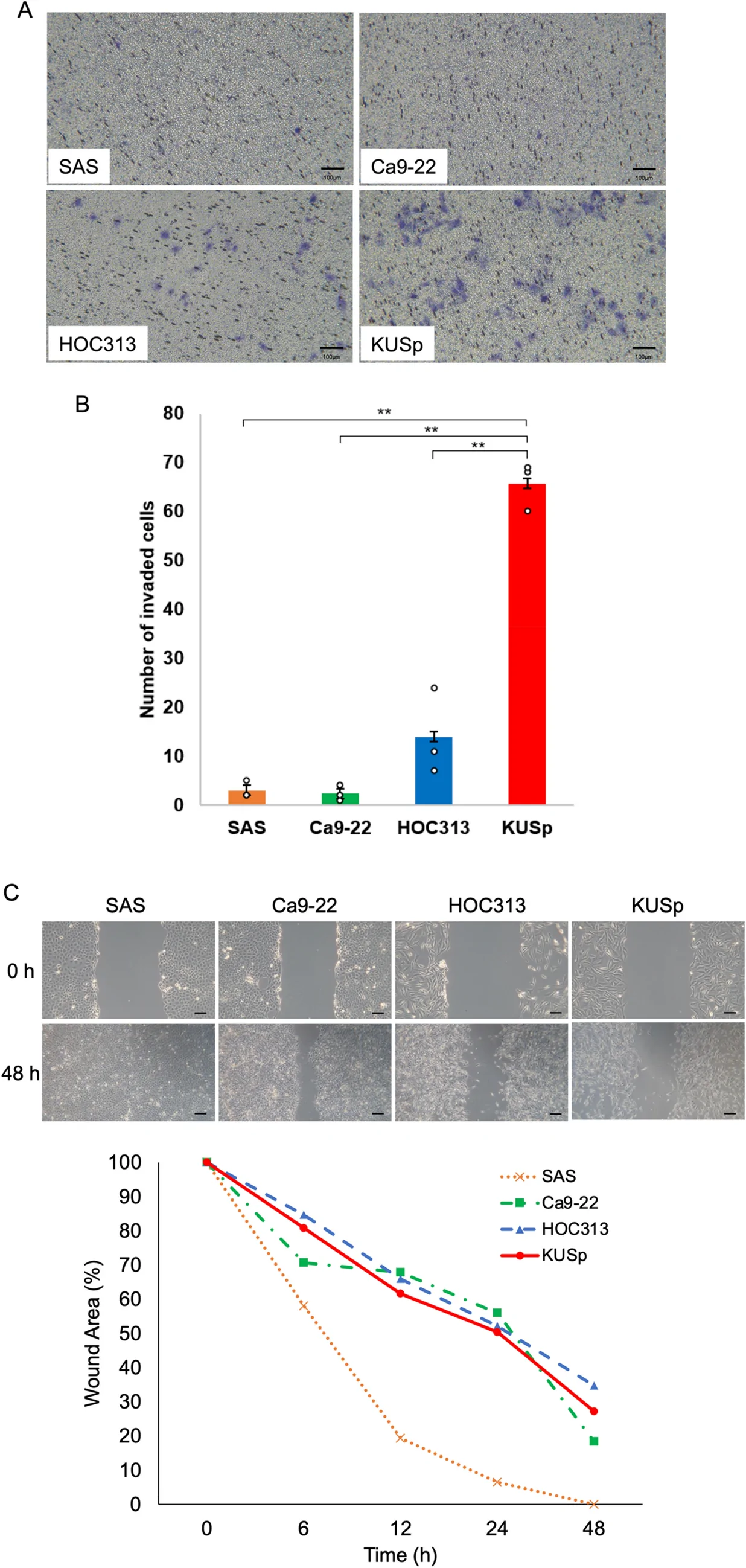
Invasion and migration abilities of KUSp and established oral squamous cell carcinoma (OSCC) cell lines. **A**, **B** Matrigel invasion assay. Representative images show invaded cells for each cell line (SAS, Ca9-22, HOC313, and KUSp) after 48 h. Quantitative analysis demonstrated that the number of invading KUSp cells was significantly higher than that of SAS, Ca9-22, and HOC313 cells. Bars represent the mean ± standard deviation (SD) of three independent experiments, and each dot represents one independent experiment (*n* = 3). Statistical significance was assessed using the Wilcoxon rank-sum (Mann–Whitney) test. **P < *0.01* for all comparisons with KUSp. **C** Scratch wound-healing assay. Representative images of wound closure at 0 and 48 h are shown for each cell line (SAS, Ca9-22, HOC313, and KUSp). Quantitative analysis shows the wound area at 48 h. KUSp cells showed wound areas comparable to those of HOC313 and Ca9-22 cells. Scale bar, 100 µm

To evaluate the invasion and migration abilities of KUSp cells, both Matrigel invasion and scratch wound healing assays were conducted using established OSCC cell lines as controls. In the invasion assay, KUSp cells demonstrated a significantly higher invasive capacity compared with the other OSCC cell lines tested (Fig. 5A). Quantitative analysis revealed that the number of invading KUSp cells was significantly greater than that of SAS and Ca9-22 cells and, notably, even higher than the highly invasive HOC313 cells [18–20] (Fig. 5B).

Similarly, in the migration assay, KUSp cells exhibited robust migratory activity. The wound closure rate of KUSp was equivalent to that observed in HOC313 cells, which are known for their high migratory ability [18–20], and was also similar to that of Ca9-22 (Fig. 5C). These results indicate that KUSp possesses both strong invasive and migratory properties, distinguishing it from other oral cancer cell lines. Taken together, these findings suggest that KUSp may serve as a valuable model for studying the mechanisms underlying tumor invasion and migration in OSCC.

### KUSp cells low expression of CAF-associated markers

**Fig. 6:**
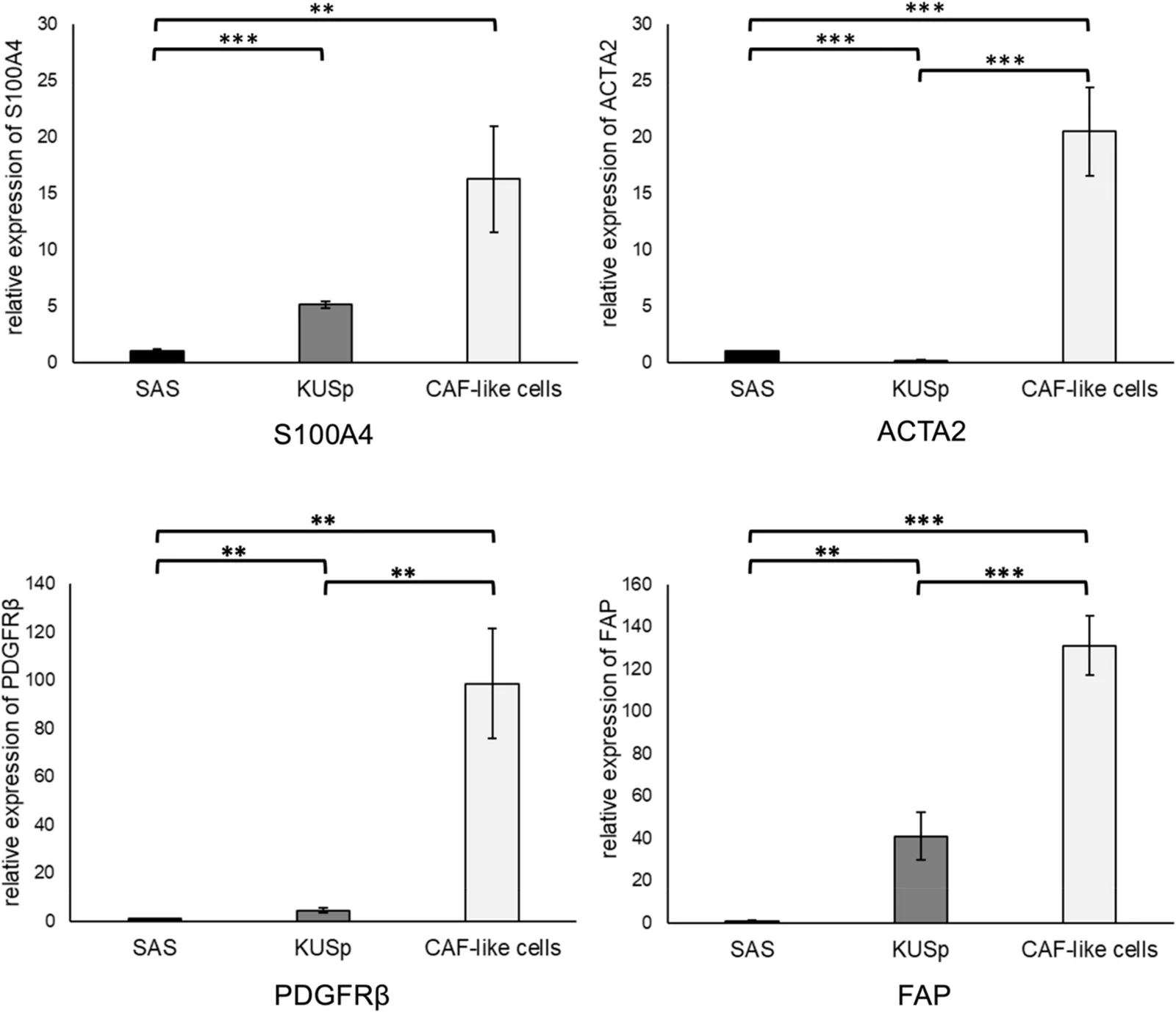
Expression of cancer-associated fibroblast-related markers in KUSp cells. Quantitative real-time PCR analysis of S100A4, ACTA2 (α-SMA), PDGFRβ, and FAP expression in SAS cells, KUSp cells, and TGF-β1-induced CAF-like cells generated from the human oral fibroblast cell line GT-1. Relative mRNA expression was normalized to GAPDH and calculated using the 2^−ΔΔCt^ method, with expression in SAS cells set to 1.0. KUSp cells showed lower expression of CAF-associated markers than TGF-β1-induced CAF-like cells, with expression levels more similar to those of SAS cells. Data are presented as the mean ± SDs from three independent experiments. **P < *0.01*, ***P < *0.001*

Given that KUSp cells exhibited a spindle-shaped morphology and EMT-like phenotype, we further evaluated whether they displayed a CAF-like transcriptional profile. Quantitative real-time PCR was performed to compare the expression of CAF-associated markers, including S100A4, ACTA2 (α-SMA), PDGFRβ, and FAP, among KUSp cells, the conventional oral squamous cell carcinoma cell line SAS, and TGF-β1-induced CAF-like cells generated from the human oral fibroblast cell line GT-1. The expression levels of S100A4, ACTA2, PDGFRβ, and FAP in KUSp cells were lower than those in TGF-β1-induced CAF-like cells and were more similar to those observed in SAS cells (Fig. 6). These results indicate that KUSp cells do not exhibit a transcriptional profile resembling that of the TGF-β1-induced CAF-like reference cells evaluated in this study.

### KUSp cells do not exhibit adipogenic or osteogenic differentiation under the tested condition

**Fig. 7:**
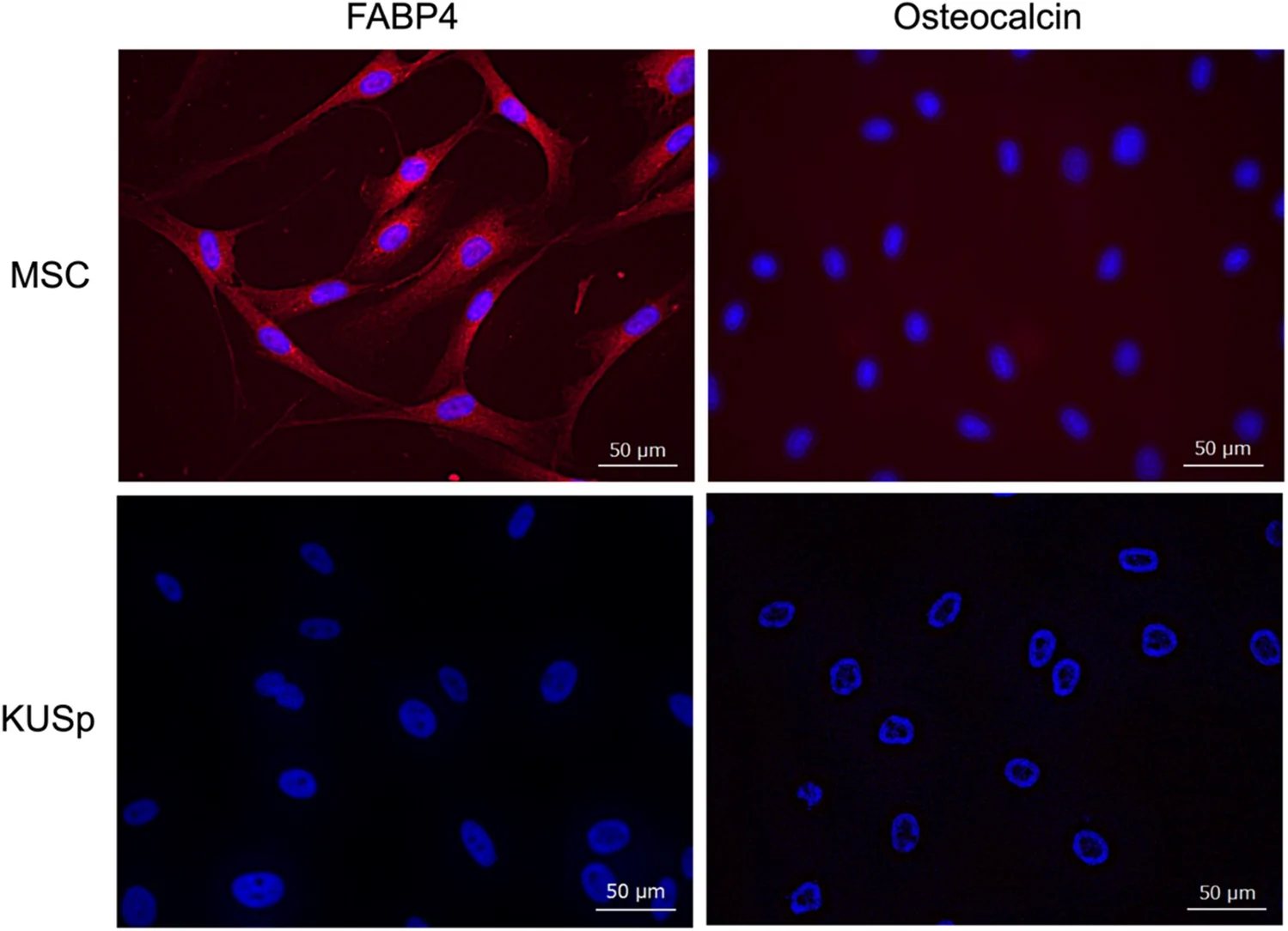
Assessment of adipogenic and osteogenic differentiation capacity of KUSp cells. Representative immunofluorescence images of FABP4 and Osteocalcin expression in adipose-derived mesenchymal stem cells (AD-MSCs) and KUSp cells cultured under adipogenic and osteogenic induction conditions, respectively. Under adipogenic induction conditions, AD-MSCs showed clear FABP4 positivity, whereas KUSp cells were negative for FABP4. Under osteogenic induction conditions, KUSp cells were negative for Osteocalcin. Osteocalcin positivity in AD-MSCs was limited under the tested conditions. Scale bars, 50 µm

To assess whether KUSp cells exhibit mesenchymal stromal/stem cell (MSC)-like differentiation capacity, KUSp cells and AD-MSCs were cultured under adipogenic and osteogenic induction conditions. Under adipogenic induction conditions, AD-MSCs used as a positive control showed clear FABP4 positivity, whereas KUSp cells were negative for FABP4. Under osteogenic induction conditions, KUSp cells were negative for Osteocalcin; however, Osteocalcin positivity in AD-MSCs was limited under the tested conditions (Fig. 7). Collectively, KUSp cells did not show evidence of adipogenic differentiation and did not express Osteocalcin under osteogenic induction conditions.

### Comparative whole-exome sequencing analysis of KUSp and the original tumor tissues

Whole-exome sequencing was performed using DNA from the primary tumor, metastatic cervical lymph node tumor, and KUSp cells. The mean sequencing depths were 108.9×, 31.0×, and 77.1×, respectively. Multiple selected variants, including TP53 p.Asp281_Thr284dup, NOTCH1 p.Cys1496Tyr, PARD3 p.Glu373*, NOX1 p.Pro70Ser, and FAT1 variants, were shared among all three specimens (Supplementary Table S3). NOTCH1 p.Cys1496Tyr showed similar VAFs in the primary tumor, metastatic tumor, and KUSp cells (28.5%, 26.7%, and 33.8%, respectively), whereas TP53 p.Asp281_Thr284dup showed marked enrichment in KUSp cells (43.8%, 52.9%, and 99.0%, respectively). Several additional variants were not evaluable in one or more specimens because of insufficient sequencing coverage.

### In vivo tumorigenicity of KUSp cells

KUSp cells (5 × 10^6^ cells/mouse) mixed with Matrigel were inoculated into nude mice and BRJ mice at dorsal subcutaneous, tongue, and buccal mucosal sites (*n* = 5 per condition). No reproducible, progressively growing tumors were observed during the 4-week observation period under any of the tested conditions (Supplementary Table S4). Therefore, histopathological comparison between KUSp-derived xenograft lesions and the original oral spindle cell squamous cell carcinoma could not be performed.

## Discussion

In this study, we successfully established and thoroughly characterized a novel human SpSCC cell line, KUSp, derived from a metastatic cervical lymph node of a patient with oral cancer. To the best of our knowledge, this is the first report describing the establishment of an SpSCC cell line from a metastatic lymph node in the context of oral cancer. The rarity of SpSCC and the technical challenges associated with establishing a cell line from metastatic lesions underscore the major scientific significance and potential utility of KUSp as a disease model.

Histopathological examination of the original tumor revealed a distinct biphasic pattern, with the superficial regions dominated by conventional SCC components and the deeper, invasive fronts exhibiting a predominance of spindle cell elements. This biphasic morphology is well recognized as a hallmark of SpSCC and has been documented in previous series of oral and head and neck spindle cell carcinoma, reflecting the histogenetic and phenotypic diversity within these tumors [1, 2, 4]. Previous clinicopathological analyses have defined the coexistence and transition between epithelial and spindle cell components as a key diagnostic feature of SpSCC and also suggested that the deeper, more invasive regions often acquire mesenchymal features associated with EMT [2, 4, 5, 21].

KUSp cells established from the metastatic cervical lymph node displayed definitive mesenchymal and EMT-related characteristics, including downregulation of epithelial markers (cytokeratin AE1/AE3 and E-cadherin) and heightened expression of mesenchymal markers (vimentin, N-cadherin, and α-SMA) (Fig. 2C). Immunofluorescence and western blotting analyses confirmed that KUSp shares an EMT-like phenotype similar to that of HOC313 (Fig. 4A, B), a known EMT-positive OSCC cell line [18–20]. Functionally, KUSp cells demonstrated significantly higher invasive capacity than conventional OSCC cell lines, including SAS and Ca9-22, and even greater invasiveness than HOC313—already recognized for its high EMT and metastatic potential [18–20]. The migratory activity of KUSp was also comparable with that observed in Ca9-22 and HOC313. These results indicate that KUSp recapitulates the highly invasive and aggressive nature typical of the spindle cell phenotype described in SpSCC and other spindle cell carcinoma. This is in line with population-based studies showing that head and neck SpSCC exhibits significantly worse disease-specific survival compared with conventional head and neck SCC [3, 9, 10].

In the primary gingival lesion, the conventional squamous component showed diffuse AE1/AE3 and E-cadherin positivity, whereas the spindle cell component exhibited only weak AE1/AE3 and E-cadherin staining together with strong vimentin expression, consistent with a partial EMT-like phenotype (Fig. 2B). In the metastatic cervical lymph node, spindle-shaped carcinoma cells remained positive for AE1/AE3 and strongly expressed E-cadherin and vimentin (Fig. 2C), indicating that epithelial characteristics were still partly preserved at the time of lymphatic dissemination despite a shift toward a more mesenchymal phenotype. Notably, E-cadherin expression was completely lost in the established KUSp cell line. This discrepancy may reflect a selection process during primary culture, in which epithelial (E-cadherin–positive) tumor cells that retained stronger epithelial features were outcompeted or eliminated, leading to the predominance of highly mesenchymal, spindle-shaped carcinoma cells in vitro. Similar shifts toward more mesenchymal phenotypes during the establishment of SpSCC or sarcomatoid carcinoma cell lines have been documented and are thought to underscore both the aggressive biology of the spindle cell component and the inherent limitations of cell line models in recapitulating the full histological heterogeneity of the original tumor [5, 11, 22]. However, from an experimental standpoint, the resulting enrichment of EMT-like tumor cells in vitro may provide a unique model system that is particularly suitable for investigating the most invasive fraction of the disease. Because KUSp cells exhibited a spindle-shaped morphology and an EMT-like phenotype, we evaluated the possibility of CAF- or MSC-like properties. KUSp cells expressed lower levels of S100A4, ACTA2, PDGFRβ, and FAP than TGF-β1-induced CAF-like cells, with an expression profile closer to that of SAS cells. In addition, KUSp cells were negative for FABP4 under adipogenic induction conditions, whereas adipose-derived MSCs used as a positive control showed clear FABP4 positivity. KUSp cells were also negative for Osteocalcin under osteogenic induction conditions; however, Osteocalcin positivity in the AD-MSCs control was limited. Although chondrogenic differentiation and flow-cytometric analysis of canonical MSC surface markers were not performed, these findings, together with the EMT-like phenotype and high invasive capacity of KUSp cells, argue against a typical CAF-like or MSC population.

Previous reports have substantially contributed to our understanding of SpSCC biology [1, 2, 4]. Kudo et al. established a human SpSCC cell line derived from the gingiva, providing a foundation for cellular studies of primary SpSCC in the oral cavity [5]. In addition, Kim et al. established a detailed analysis of an SpSCC cell line, offering comparative morphological and molecular insights between SpSCC and conventional SCC and highlighting the significance of EMT marker expression and biological aggressiveness in SpSCC [11]. Nevertheless, such reports remain limited, specifically with regard to well-characterized SpSCC cell lines derived from metastatic lesions [3]. In this study, we established and characterized a novel SpSCC cell line, KUSp, derived from a metastatic cervical lymph node, thus providing a valuable patient-derived in vitro model for investigating the molecular mechanisms associated with spSCC progression. Comparative analysis between primary tumor–derived and metastatic SpSCC cell lines may yield insights into the evolution and selection of aggressive tumor phenotypes and expand available resources for SpSCC research [5, 11]. The STR profile of KUSp was compatible with those of the corresponding primary tumor and metastatic cervical lymph node tumor and was distinct from those of both HOC313 and HSC-2 (Supplementary Table S2). Comparative whole-exome sequencing further supported the genetic relationship between KUSp and the original tumor tissues (Supplementary Table S3). Multiple selected variants, including TP53 p.Asp281_Thr284dup, NOTCH1 p.Cys1496Tyr, PARD3 p.Glu373*, NOX1 p.Pro70Ser, FOSL2 p.Lys239fs, STARD7 p.Ser53Leu, TARBP1 p.Ser1469Leu, and FAT1 variants, were shared among the primary tumor, metastatic cervical lymph node tumor, and KUSp cells. The NOTCH1 p.Cys1496Tyr variant showed similar variant allele frequencies across the three specimens, whereas TP53 p.Asp281_Thr284dup showed marked enrichment in KUSp cells. These findings support genomic continuity between KUSp and the original tumor tissues and suggest that specific tumor subclones may have been enriched during metastatic progression and/or cell-line establishment and subsequent in vitro culture. However, variant allele frequencies can be influenced by tumor purity, copy-number alterations, and allelic imbalance. In addition, because several loci were not evaluable owing to insufficient sequencing coverage, the present comparison cannot definitively distinguish clonal selection from culture-associated genomic changes or establish complete genomic concordance. Accordingly, the shared variants support the patient-tumor origin of KUSp, while the in vitro phenotype should be interpreted with consideration of possible clonal selection during establishment and culture.

Taken together, our findings indicate that KUSp is a patient-derived in vitro model of metastatic oral SpSCC, enabling studies of EMT-associated phenotypes, invasion, and migration. KUSp may serve as a useful in vitro experimental resource for mechanistic studies of oral spSCC progression. A limitation of this study is that KUSp cells did not reproducibly form tumors in vivo under the conditions examined. Despite inoculation of KUSp cells mixed with Matrigel into nude mice and BRJ mice at dorsal subcutaneous, tongue, and buccal mucosal sites, no reproducible, progressively growing tumors were observed during the 4-week observation period (Supplementary Table S4). Consequently, we could not determine whether KUSp-derived xenograft lesions recapitulate the histopathological features of the original oral SpSCC. The inability of conventional cell culture models to fully reproduce tumor architecture and microenvironmental interactions, together with the variable engraftment of HNSCC models in vivo, remains an important limitation of preclinical modeling [23]. Therefore, KUSp should primarily be regarded as an in vitro model for investigating EMT-associated phenotypes and invasion- and migration-related properties relevant to oral SpSCC progression.

In conclusion, we established and characterized KUSp, a patient-derived in vitro cell-line model of metastatic oral spindle cell squamous cell carcinoma. The EMT-associated, migratory, and invasive features of KUSp support its use for mechanistic studies of oral SpSCC progression. Although reproducible in vivo tumor formation was not achieved under the conditions tested, KUSp may provide a useful in vitro experimental resource for investigating EMT-associated metastatic progression and for exploratory evaluation of candidate therapeutic vulnerabilities in this rare malignancy.

## Supplementary Information

Below is the link to the electronic supplementary material.

Supplementary file1 (XLSX 12 kb)

Supplementary file2 (docx 83 kb)

Supplementary file3 (docx 26 kb)

## Data Availability

The datasets used and/or analyzed during the current study are available from the corresponding author upon reasonable request.
