# Supplementary material for: KUSp, a novel oral spindle cell squamous cell carcinoma cell line with epithelial-mesenchymal transition features and high invasive capacity
Source: Hum Cell. 2026 Nov 7;39(11):156. doi: 10.1007/s13577-026-01464-3 (PMC13744505; doi:10.1007/s13577-026-01464-3)
Supplement: Supplementary file 1 [file 13577_2026_1464_MOESM2_ESM.docx]

**Supplementary Table S1. Primer sequences used for RT-qPCR**

| Supplementary Table S1. Primer sequences used for RT-qPCR | |
| --- | --- |
| Gene | Primer Sequence (5'–3') |
| FAP | Forward: 5'- TGAACGAGTATGTTTGCAGTGG-3' |
|  | Reverse: 5'- GGTCTTTGGACAATCCCATGT-3' |
| PDGFRβ | Forward: 5'- TGATGCCGAGGAACTATTCATCT-3' |
|  | Reverse: 5'- TTTCTTCTCGTGCAGTGTCAC-3' |
| S100A4 | Forward: 5'- GATGAGCAACTTGGACAGCAA-3' |
|  | Reverse: 5'- CTGGGCTGCTTATCTGGGAAG-3' |
| ACTA2 | Forward: 5'- AAAAGACAGCTACGTGGGTGA-3' |
|  | Reverse: 5'- GCCATGTTCTATCGGGTACTTC-3' |
| GAPDH | Forward: 5'- TGTTGCCATCAATGACCCCTT-3' |
|  | Reverse: 5'- CTCCACGACGTACTCAGCG-3' |

**Supplementary Table S2. Short tandem repeat profiles of the primary tumor, metastatic cervical lymph node tumor, KUSp, HOC313, and HSC-2 cells.**

| Supplementary Table S2. Short tandem repeat profiles of the primary tumor, metastatic cervical lymph node lesion, KUSp, HOC313, and HSC-2 cells. | | | | | | | | | | |  |
| --- | --- | --- | --- | --- | --- | --- | --- | --- | --- | --- | --- |
|  |  |  |  |  |  |  |  |  |  |  |  |
| Locus | Primary tumor | | Metastatic cervical lymph node lesion | | **KUSp** | | HOC313 | | HSC-2 | |  |
|  |  |  |  |  |  |  |  |  |  |  |  |
| D3S1358 | 16 | 18 | 16 | 18 | 16 | 18 | 15 | 18 | 15 | 17 |  |
| TH01 | 7 | 9 | 7 | 9 | 7 |  | 7 |  | 6 | 7 |  |
| D21S11 | 29 |  | 29 |  | 29 |  | 30 | 32.2 | 31.2 |  |  |
| D18S51 | 15 | 16 | 16 |  | 15 | 16 | 17 |  | 19 |  |  |
| Penta_E | 5 | 11 | Not detected |  | 5 | 11 | 14 |  | 12 | 15 |  |
| D5S818 | 9 | 10 | 9 | 10 | 9 | 10 | 13 |  | 10 | 12 |  |
| D13S317 | 8 | 9 | 8 | 9 | 8 | 9 | 8 | 12 | 11 | 12 |  |
| D7S820 | 10 | 11 | 10 | 11 | 10 | 11 | 10 | 11 | 9 | 12 |  |
| D16S539 | 9 | 10 | 10 |  | 9 | 10 | 11 | 12 | 12 |  |  |
| CSF1PO | 12 |  | 12 |  | 12 |  | 11 |  | 12 | 13 |  |
| Penta_D | 10 | 11 | Not detected |  | 10 |  | 9 | 11 | 9 |  |  |
| AMEL | X |  | X |  | X |  | X |  | X |  |  |
| vWA | 18 | 19 | 18 | 19 | 18 | 19 | 14 | 18 | 16 | 18 |  |
| D8S1179 | 10 |  | 10 |  | 10 |  | 10 | 15 | 10 | 14 |  |
| TPOX | 11 |  | 11 |  | 11 |  | 8 |  | 8 |  |  |
| FGA | 23 |  | 23 |  | 23 |  | 19 | 22 | 19 | 21 |  |
| “Not detected” indicates loci for which no allele was detected in the metastatic lesion. AMEL, amelogenin. | | | | | | | | | | |  |

**Supplementary Table S4. Summary of in vivo tumorigenicity experiments using KUSp cells.**

| Supplementary Table S4. Summary of in vivo tumorigenicity experiments using KUSp cells | | | | | | |  |
| --- | --- | --- | --- | --- | --- | --- | --- |
| Mouse  strain | Implantation site | KUSp cells  per mouse | Matrigel | Mice  per group | Observation  period | Tumor  formation |  |
| Nude | Dorsal subcutaneous tissue | 5 x 10^6^ | Yes | 5 | 4 weeks | 0/5 |  |
| Nude | Tongue | 5 x 10^6^ | Yes | 5 | 4 weeks | 0/5 |  |
| Nude | Buccal mucosa | 5 x 10^6^ | Yes | 5 | 4 weeks | 0/5 |  |
| BRJ | Dorsal subcutaneous tissue | 5 x 10^6^ | Yes | 5 | 4 weeks | 0/5 |  |
| BRJ | Tongue | 5 x 10^6^ | Yes | 5 | 4 weeks | 0/5 |  |
| BRJ | Buccal mucosa | 5 x 10^6^ | Yes | 5 | 4 weeks | 0/5 |  |
| Abbreviations: BRJ, BALB/c-Rag2^-/-^Jak3^-/-^; Matrigel, basement membrane matrix KUSp cells were mixed with Matrigel before inoculation. No progressively growing tumors were observed during the 4-week observation period. | | | | | | |  |
|  |  |  |  |  |  |  |  |
