# Supplementary material for: KUSp, a novel oral spindle cell squamous cell carcinoma cell line with epithelial-mesenchymal transition features and high invasive capacity
Source: Hum Cell. 2026 Nov 7;39(11):156. doi: 10.1007/s13577-026-01464-3 (PMC13744505; doi:10.1007/s13577-026-01464-3)
Supplement: Supplementary file 2 [file 13577_2026_1464_MOESM3_ESM.docx]

**Appendix S1. Whole-exosome sequencing and comparative variant analysis**

**Library preparation and whole-exome sequencing**
Genomic DNA extracted from primary tumor tissue (Primary), metastatic lymph node tissue (Meta), and the established KUSp cell line was subjected to whole-exome sequencing (WES). Target enrichment was performed using the SureSelect Human All Exon V6 (58 Mb) kit (Agilent Technologies, Santa Clara, CA, USA). Libraries were sequenced using 150-bp paired-end reads on an Illumina NovaSeq 6000 platform (Illumina, San Diego, CA, USA).

**Pre-processing and sequence alignment**
The quality of paired-end FASTQ files was assessed using FastQC (v0.11.7). Adapter sequences and low-quality bases were removed using Trimmomatic (v0.38) with the following parameters: ILLUMINACLIP:<adapter.fa>:2:30:10 LEADING:20 TRAILING:20 SLIDINGWINDOW:4:15 MINLEN:36. Trimmed reads were aligned to the human reference genome GRCh38/hg38 using BWA-MEM (v0.7.17-r1188). SAM files were converted to BAM files and sorted using Picard SortSam (v2.18.11). PCR duplicates were marked using Picard MarkDuplicates. Local realignment around known indels and base quality score recalibration were performed using GATK (v4.0.8.1), with dbSNP138 used as a known-sites resource. The recalibration table was applied using GATK ApplyBQSR.

**Variant calling and annotation**
Single-nucleotide variants (SNVs) and short insertions/deletions (indels) were identified using GATK HaplotypeCaller (v4.0.8.1) in GVCF mode. Variants were annotated using SnpEff (v4.3t) for predicted functional effects. Clinical significance and population frequency annotations were obtained from ClinVar, the GWAS Catalog, the 1000 Genomes Project, InterPro, and dbNSFP. Functional prediction scores from dbNSFP, including CADD, FATHMM, LRT, MetaSVM, MutationAssessor, MutationTaster, PROVEAN, PolyPhen-2, SIFT, and phastCons, were used to support interpretation of non-synonymous variants.

**Comparative analysis of variants among Primary, Meta, and KUSp**
Variant profiles of Primary, Meta, and KUSp were integrated and compared to characterize the genomic features of KUSp and to assess concordance with the original tumor tissues. Shared variants were defined by identical chromosome, genomic position, reference allele, and alternate allele. For each variant, reference-allele read count, alternative-allele read count, sequencing depth, and variant allele frequency (VAF) were recorded for each specimen. VAF was calculated as the proportion of alternative-allele reads among total reads at the corresponding locus. Variants with insufficient sequencing coverage in one or more specimens were classified as not evaluable; lack of a detected variant in such samples was not interpreted as its true absence.
